# Protease-activated receptor 1 as a potential therapeutic target for COVID-19

**DOI:** 10.1177/1535370220978372

**Published:** 2020-12-10

**Authors:** Emanuel S. Rovai, Tomaz Alves, Marinella Holzhausen

**Affiliations:** 1 Department of Dentistry, University of Taubate, Taubate 12010-490, Brazil; 2 Division of Periodontics, Department of Stomatology, School of Dentistry, University of São Paulo, São Paulo 05508-000, Brazil

**Keywords:** Protease-activated receptor 1, COVID-19, coronavirus, proteases, coagulopathy, anticoagulation

## Abstract

Acute respiratory disease caused by a novel coronavirus (SARS-CoV-2) has spread all over the world, since its discovery in 2019, Wuhan, China. This disease is called COVID-19 and already killed over 1 million people worldwide. The clinical symptoms include fever, dry cough, dyspnea, headache, dizziness, generalized weakness, vomiting, and diarrhea. Unfortunately, so far, there is no validated vaccine, and its management consists mainly of supportive care. Venous thrombosis and pulmonary embolism are highly prevalent in patients suffering from severe COVID-19. In fact, a prothrombotic state seems to be present in most fatal cases of the disease. SARS-CoV-2 leads to the production of proinflammatory cytokines, causing immune-mediated tissue damage, disruption of the endothelial barrier, and uncontrolled thrombogenesis. Thrombin is the key regulator of coagulation and fibrin formation. In severe COVID-19, a dysfunctional of physiological anticoagulant mechanisms leads to a progressive increase of thrombin activity, which is associated with acute respiratory distress syndrome development and a poor prognosis. Protease-activated receptor type 1 (PAR1) is the main thrombin receptor and may represent an essential link between coagulation and inflammation in the pathophysiology of COVID-19. In this review, we discuss the potential role of PAR1 inhibition and regulation in COVID-19 treatment.

## Impact statement

This minireview brings attention to the key role of the protease-activated receptor 1 (PAR1) in the pathophysiological outcomes derived from the SARS-CoV-2 infection, focusing on the coagulatory and inflammatory aspects. Further, we addressed therapeutic strategies targeting PAR1 blockage and how they may present a potential as an effective treatment strategy to prevent further progression of the coagulopathy and inflammatory dysfunctions associated with the COVID-19.

## Introduction

Coronavirus disease 2019 (COVID-19) is a current pandemic provoked by the severe acute respiratory syndrome coronavirus 2 (SARS-CoV-2). COVID-19 has been impacting 188 countries and territories, being reported to affect around 50,517,420 people worldwide, as of 9 November 2020, as reported by the Center for Systems Science and Engineering at John Hopkins University.^1^

SARS-CoV-2 virus affects the respiratory system, leading to symptoms including fever, gastrointestinal symptoms, general weakness, shortness of breath, dry cough, headache, and dizziness.^2^ In fact, complications of COVID-19 are highly diversified, going from pneumonia to sepsis,^3^ and respiratory failure with acute respiratory distress syndrome (ARDS).^4^ This disease can be fatal, and over 1 million global deaths have been reported.^1^

Current therapy is mainly adjuvant, with no approved vaccine against COVID-19 still available. Several drugs, including antivirals and antimalarial/parasiticide drugs, have been tested in clinical trials, but none of them have been certified to be a precise therapeutics yet.^5^ Here we will review some insights with regards to the pathogenesis of COVID-19 in severe cases and consider the protease-activated receptor 1 (PAR1) as a potential therapeutic target.

## Association of coagulopathic disorder in severe SARS-CoV-2 infection

The levels of proinflammatory cytokines and procoagulation factors increase according to the SARS-CoV2 disease severity. In mild COVID-19, there is a low-grade inflammation profile corresponding to a normal immunological response against viral infection, with moderately enhanced levels of interferon-γ, tumor necrosis factor (TNF)-α, interleukin (IL)-1, IL-6, and IL-2.^6^ These cytokines increase local procoagulant responses by upregulating tissue factor (TF) VIII and von Willebrand factor (vWF), and downregulating antithrombin (AT).^7^ In severely ill patients, the amplification of inflammatory responses, known as cytokine release syndrome (CRS) is characterized by the massive release of proinflammatory cytokines, and may be associated with acute lung injury (ALI) or ARDS.^6^ Eventually, CRS may become uncontrolled and linked to a clinical phenotype that resembles a form of secondary hemophagocytic lymphohistiocytosis or macrophage activation syndrome in critical-COVID-19 patients.^8,9^ This complication triggers a marked elevation of procoagulant factors and decrease of natural coagulation inhibitor levels and is often linked to multiple organ failure (MOF) and death.

Acute thrombotic events, mainly venous thrombosis and pulmonary embolism are highly prevalent in patients suffering from severe COVID-19.^10,11^ Histological data have also demonstrated the role of a widespread microthrombosis in the pathogenesis of organ debilitation during SARS-CoV-2 infection,^12,13^ which could be in favor of a prothrombotic state originally in the lung that can disseminate throughout the body.

The sepsis-induced coagulopathy (SIC), which is a prothrombotic state, precedes disseminated intravascular coagulation (DIC).^14^ SIC and/or DIC are present in most mortal cases of COVID-19 marked by organs dysfunction, such as ARDS.^8,15–17^ Inflammatory cytokines are crucial mediators of the coagulation disorders during sepsis.^3^ In fact, the severe form of COVID-19 is characterized by a marked inflammatory response, described by expressive influx of leukocytes into the lungs,^18^ and increased serum levels of proinflammatory cytokines, which is in line with the concept of “cytokine storm”.^19^ Interestingly, inflammation leads to coagulation, and coagulation pathways activation leads to further inflammation by enhancing the production of proinflammatory cytokines and possibly leading to MOF.^20^ In this model, activated platelets, immune cells, fibrin, and coagulation proteases may promote this bidirectional relationship.^21^ The resulting immune thrombotic response initially may be considered as an action to avoid spread of SARS-CoV-2 outside the alveoli by promoting pathogen recognition and functioning as an obstacle to avoid more virus assault, but later it can turn uncontrolled, leading to vascular and tissue damage.^22^

SARS-CoV-2 directly infects epithelial and endothelial cells, resulting in the generation of inflammatory mediators, causing immune-mediated endothelial barrier, and tissue breakdown, and thrombogenesis.^23^ These thromboinflammatory changes have been observed not only in the lungs,^24^ but also in the intestine, liver, kidneys, and heart of COVID-19 patients.^25–28^

During this process, D-dimer levels are increased as a result of fibrin degradation from residual tissue plasminogen activator/plasmin activity, and from human neutrophil elastase activity. In fact, several studies reported that serious cases of SARS-CoV-2 infection present significantly increased D-dimer, longer prothrombin time, activated partial thromboplastin time, increased vWF activity and vWF antigen, as well as factor VIII, and plasminogen activator inhibitor 1 (PAI-1).^11,17,26,29,30^ Moreover, increased levels of thrombin-AT complexes have also been reported, thus suggesting that thrombin may be highly active in seriously ill COVID-19 patients.^31^

Thrombin is a key regulator of coagulation and fibrin formation, and it is generated by TF coupled with a dysfunctional fibrinolytic response caused by PAI-1.^32^ Initially, a small amount of thrombin is produced by the activation of TF. Then, thrombin activates the factor X amplifying thrombin formation, leading to the production of fibrin from fibrinogen. Under normal circumstances, thrombin generation is controlled by physiological anticoagulant pathways, such as AT III, TF pathway inhibitor, and the activated protein C (APC) system.^33^ In severe COVID-19, all these control mechanisms can be dysfunctional, with a progressive increase of thrombin activity being associated with ARDS development and a poor prognosis.^17,34^

PAR1 is the major thrombin receptor intermediating thrombin-induced platelet aggregation^35^ and may represent a key association between coagulation and inflammation in the pathophysiology of COVID-19.

## Protease-activated receptor 1 (PAR1)

Infectious complications such as COVID-19 are associated with coagulopathy. Systemic host inflammatory response leads to an increased release of proinflammatory cytokines, which have pleiotropic effects, including activation of coagulation.^36^ Besides, it has become evident that there is a cross-talk between coagulation molecules and inflammatory mediators, indicating that coagulation also influences inflammation.^37,38^ Coagulation molecules binding to PARs represent the primary mechanism involved in this process.^35^

PARs belong to the family of the G-protein coupled receptors. Their activation occurs through proteolytic cleavage of the N-terminal domain by proteinases, resulting in the generation of a new N-terminal that binds to the receptor itself, and its autoactivation.^39^ So far, four members of the PAR family have been discovered, PAR1, PAR2, PAR3, and PAR4.^40^ Since PAR1, previously known as the thrombin receptor, plays a significant role in coagulation and inflammation, a high interest arose on its involvement in the hypercoagulable state and ischemia associated with SARS-CoV-2. PAR1 is expressed by endothelial cells^41,42^ and lung cells.^43^ It is highly expressed in both intra- and extravascular compartments of the injured lung, being associated with endothelial dysfunction and a prothrombotic state.^35,39,42^ In fact, in lung tissue, additionally to endogenous enzymes (i.e., thrombin, matrix metalloproteinase-1 (MMP-1), and APC), PAR1 activity can also be regulated by exposed pathogen-derived proteinases, playing both pro and anti-inflammatory roles depending on the activator. PAR2 is expressed by several cells (i.e., bronchial and alveolar epithelial cells, neutrophils, monocytes, fibroblasts, and osteoblasts) and plays an important role in host immune defense and lung inflammation.^39,44^ The main activator of PAR2 is trypsin, although other endogenous proteinases, including coagulation factors and pathogen-derived proteinases, can also activate it. PAR3 and PAR4 can be activated by thrombin.^39^ In fact, it is suggested that PAR3 may act as cofactor for thrombin-mediated activation of PAR4. Further, while PAR1 mediates human platelets' activation at low thrombin concentrations, PAR4 requires a high concentration to trigger platelet aggregation and secretion.^35^

**Figure 1.**
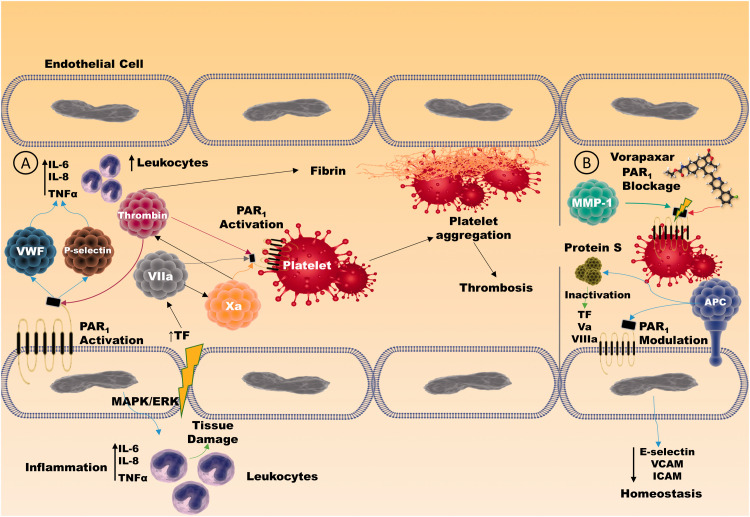
PAR1 functions in thrombotic and inflammatory processes in the vascular endothelium. (a) PAR1 activation by thrombin and factors VIIa and Xa induces an intracellular response in endothelial cells and platelets through the ERK1/2 on the canonical pathway, producing a series of proinflammatory and prothrombotic mediators such as P-selectin and vWF that lead simultaneously to thrombosis and tissue inflammation in a crosslink process. (b) PAR1 therapeutic blockage through Vorapaxar® and/or modulation via APC-induced activation produces a non-canonical pathway signaling which impairs inflammatory and prothrombotic endothelial outcomes. Moreover, APC directly participates in the anticoagulatory processes by binding to protein S, further inactivating TF, Va, and VIIIa. (A color version of this figure is available in the online journal.)

PAR1 is considered the primary mediator of thrombin-stimulated platelet aggregation and clot formation.^35^ Thrombin cleaves PAR1 at the Arg41 site, which induces a proinflammatory response in endothelial cells, resulting in the disruption of the endothelial barrier function, all these mediated through ERK1/2 phosphorylation and subsequent RhoA signaling pathway.^45,46^ Negative feedback loops and physiological anticoagulants regulate thrombin generation. During sepsis-induced inflammation, there is a breakdown in the procoagulant–anticoagulant balance, which predisposes to the formation of microthrombosis and SIC, conditions associated with severe COVID-19 features. PAR1 activation by thrombin leads to a release of P-selectin and vWF, providing an essential link between thrombosis and inflammation^47^ (Figure 1). In endothelial cells, thrombin can increase platelet-activating factor production, which acts as a potent neutrophil stimulator and can increase the release of proinflammatory cytokines such as IL-6 and 8.^47^ Importantly, this developed proinflammatory state can further potentialize thrombotic responses in the endothelium since cytokines (such as TNF-α), and pathogen products can induce even more thrombin generation.^48,49^ Still, TF represents one of the most critical sources of thrombin generation during sepsis state. Furthermore, TF VIIa and factor Xa trigger PAR1, also contributing to the development of a proinflammatory state and microthrombosis through platelet aggregation and endothelium dysfunction^36^ (Figure 1).

On the other hand, PAR1 activation by APC results in the generation of a different tethered ligand that protects endothelial cells from inflammatory biomarkers. APC cleaves PAR1 at the noncanonical site (Arg46) in the endothelium and leads to antiapoptotic stimuli and barrier function fortifying^46,50,51^ (Figure 1). APC activates signaling pathways that inhibit NFκB, decreasing proinflammatory cytokines production, and downregulate endothelial cell adhesion molecules such as ICAM, VCAM, and E-selectin.^52^ In addition to its anti-inflammatory capacity, APC can act as an anticoagulant factor through its dissociation from the membrane-surface and binding to protein S, inactivating TFs Va and VIIIa, essential cofactors for thrombin generation and hence blood clot cascade.^53^

PAR1 has been implicated in developing several respiratory diseases, such as ARDS, because of its role in coagulation and inflammation. Indeed, PAR1 primary activator (thrombin) levels are found to be increased in chronic lung injury, participating not only in the coagulatory processes but also exerting a profibrotic cellular effect.^45,54^ In the inflammation of the respiratory airways, there is an overexpression of MMPs, which has been implicated in ARDS's pathogenesis, especially for its collagenolytic (MMP-1 and -8) and gelatinolytic (MMP-2 and -9) activities.^55^ MMP-1 triggers PAR1 via biased signaling and contributes to arterial thrombosis. In a sepsis state, MMP-1 levels are increased in plasma and highly correlated with endothelial barrier disruption, DIC, lung vascular permeability, and the CRS.^56^

PAR1 has been investigated in host immunity to other viral infections, such as coxackie virus, influenza A virus, paramyxovirus, dengue virus, herpes simplex virus, and HIV. These studies have suggested that a viral infection triggers thrombin production and that PAR1-resulted activation may be a risk factor in vascular pathology associated with disease severity.

## Proteases play key roles in the SARS-CoV-2 machinery

SARS-CoV-2 is formed by RNA-dependent RNA polymerase and constitutional proteins, such as transmembrane spike (S) glycoprotein, which is used for recognition of host cell receptors, anchor, and cell invasion.^57^ The S protein involves two functional subunits, and it is cleaved at the S1/S2 multibasic cleavage site by the subtilisin-like host cell protease furin.^58^ Furin is one of the proproteins of the convertase family and is used in the maturation of viral glycoprotein of coronaviruses.^59^ Interestingly, in a murine model of severe human metapneumovirus (hMPV) lung infection, PAR1 inhibition reduced furin-mediated cleavage of the hMPV fusion protein, probably by decreasing furin levels and/or activity.^60^ Since furin has been potentially associated with the pathogenesis of SARS-CoV-2 and with the increased rates of transmission, it has been considered a potential therapeutic target.^61^

The S1 subunit of SARS-CoV-2 binds to its target, the receptor angiotensin-converting enzyme 2 (ACE2), which is connected to the external surface of the membrane of endothelial and epithelial cells.^62,63^ Binding to ACE2 leads to NFκB-driven inflammation, and proinflammatory cytokines overproduction,^64^ which can cause vasculature damage and thrombosis, and consequent TF and thrombin production. Exogenous recombinant ACE2 has been shown to be efficient in animal models of ALI/ARDS^62,65^ and has been investigated as a possible preventive therapy to COVID-19 pneumonia. It is believed that this soluble form of ACE2 may function as a decoy receptor that could bind to SARS-CoV-2, thus inhibiting membrane-bound ACE2-mediated virus entry.

The S2 subunit, which encompass the fusion system, is divided by the transmembrane serine protease 2 (TMPRSS2), a protease manifested by epithelial cells of the respiratory tract.^66^ This serine protease with trypsin-like activity is then released into the extracellular space, where it may activate the membrane bound PAR2, which is expressed by several cells in the lung and is involved in inflammation.^67^ Recently, nafamostat mesylate and camostat mesylate, TMPRSS2 inhibitors,^34,66^ have currently been studied in patients with COVID-19. These protease inhibitors have been approved for the therapeutic management of chronic pancreatitis and post-operative reflux esophagitis.

In addition, the replication machinery of SARS-CoV-2 relies on its main protein (Mpro), a 3-chymotrypsin-like protease that is crucial to process the polyproteins translated from the viral RNA.^68^ The crystallography structure of SARS-CoV-2 Mpro reveals high similarity with human coagulation factors, thrombin, and factor Xa,^69^ which may suggest a perturbation in pro-clotting coagulation by COVID-19 through PAR1 activation. Interestingly, preliminary comparison docking analysis has shown that apixaban, a factor Xa inhibitor, seems to have more affinity to SARS-CoV-2 MPro than argatroban, a direct thrombin inhibitor.^69^

Moreover, the ALI launches a cascade of proinflammatory mediators that induce alveolar macrophages and neutrophils to release MMPs. It was demonstrated that in individuals with lung disease and measurable levels of MMP-1 and/or MMP-3, there is a much higher occurrence of complications by MOF.^70^ In addition, an *in vitro* study showed that MMPs are key regulators of cell fusion and viral replication in murine coronaviruses.^71^ Tetracycline derivatives such as doxycycline, MMP inhibitor, have also been considered for COVID-19 therapy, as they have both antiviral and anti-inflammatory effects.^72^

Taken together, these findings highlight a key role for SARS-CoV-2 associated proteolytic activity during infection. Although protease inhibition seems to be appealing in COVID-19 treatment, it has to be cautiously evaluated as it may lead to some unpredictable side effects because of complex functions played by proteases in normal physiological processes. Since PAR1 activation is the essential step mediating coagulopathic disorder and immune activation, its inhibition may play a more interesting therapeutic approach as it may modulate thrombosis, DIC, and MOF associated with severe COVID-19 pneumonia.

## A possible role of PAR1 regulation in COVID-19 treatment

Considering the existing evidence on venous thromboembolism (VTE) treatment and prophylaxis, antithrombotic therapies have been suggested in the management of coagulopathy in severe COVID-19 patients.^73–75^ Among the antithrombotic agents, heparins have less drug interactions with potential antiviral medication if compared to antiplatelets and direct oral anticoagulants.^76^ Although valid scientific evidence scarcity, prophylactic doses of low molecular weight heparin (LMWH) have been recommended for all COVID-19 patients.^77^ In addition to its anticoagulant effect mediated by the activation of circulating endogenous AT, heparin also exerts anti-inflammatory,^78^ and antiviral activities.^79^ Currently, several randomized clinical trials of LMWH have been conducted to compare the use of intermediate or therapeutic heparin doses in COVID-19 patients.^80^ However, it seems reasonable to believe that LMWH may not be effective, since the severe inflammatory response in COVID-19 is possibly associated with markedly decreased levels of AT.^38,77^ Indeed, a retrospective study found a high incidence rate of VTE in mechanically ventilated severe COVID-19 patients even under prophylactic or therapeutic anticoagulation use of heparin.^81^

Because PAR1 acts on the coagulation system independently of the presence of endogenous anticoagulants, PAR1 antagonism could be considered as an attractive target to block platelet aggregation, and potentially protect against cardiovascular diseases, thromboembolism,^82^ post-ARDS pulmonary fibrosis,^83^ and pulmonary edema in critical COVID-19 pneumonia.^80^ Vorapaxar is a clinically approved PAR1competitor antagonist that binds to the receptor, competing with endogenous agonists, preventing thrombin-mediated platelet activation.^84^ Clinical trials demonstrated that vorapaxar is associated with a 12% reduced rate of cardiovascular events such as myocardial infarction and stroke.^82^ Besides, *in vivo* studies demonstrated that a PAR1 antagonist (SCH79797) could decrease the levels of several inflammatory biomarkers, neutrophilic lung inflammation, and alveolar leak during bacterial pneumonia.^85^ However, it should be pointed out that vorapaxar has been used in combination with aspirin and other antiplatelet drugs. As expected with antiplatelet therapy, clinical studies demonstrated increased bleeding in patients treated with vorapaxar.^84^ The half-life of vorapaxar is expected to be too prolonged in the management of acute illness, notably considering the difficulty to reverse its bleeding risk. PAR1 antagonists such as RWJ58259 have short half-lives and could be revisited since they never progressed to clinical trials. Interestingly, PAR1 antagonization through topic delivery of Ankaferd Blood Stopper is currently being presented as a potential treatment for oral mucositis presented in COVID-19 as this drug activates a cascade of pleiotropic effects on cell proliferation, endothelial cells and angiogenesis as well as exerts anti-inflammatory and direct anti-viral effects against SARS-CoV strains.^86^

The anticoagulant, anti-inflammatory, and barrier protective effects of APC put it as a possible PAR1 modulator agent that could be explored in COVID-19 treatment. Recombinant APC was the first agent approved for human use, capable of stimulating PAR1-mediated cytoprotection in severe sepsis.^87^ However, because of APC bleeding liability, variants have been created based mainly on its cytoprotection, with limited anticoagulant activity.^88^ Clinical studies have been conducted to verify its safety and effectiveness in treating several conditions, including ischemic stroke.

Taken together, PAR1 may represent an important target for the treatment and prevention of COVID-19 complications, either by its inhibition and consequent anti-inflammatory, antithrombotic, and antifibrotic activities or through its modulation and cytoprotective activity.
